# Longitudinal network analysis of depression, anxiety, and post-traumatic stress disorder comorbidities among adolescents in regional China

**DOI:** 10.3389/fpubh.2025.1522877

**Published:** 2025-03-17

**Authors:** Heting Li, Jiahe Liu, Yamin Wang, Zhenchao Li, Shiwei Mei, Zigang Zhang, Linlin Fan, Lihua Jiang

**Affiliations:** ^1^Department of Health Policy and Management, West China School of Public Health/West China Fourth Hospital, Sichuan University, Chengdu, China; ^2^AIM for Health Lab, Faculty of Information Technology, Monash University, Melbourne, VIC, Australia; ^3^Teaching & Research Section of General Practice, The General Practice Medical Center, West China Hospital of Sichuan University, Chengdu, China

**Keywords:** longitudinal study, network analysis, post-traumatic stress disorder, depression, anxiety, adolescent health, COVID-19, comorbidities

## Abstract

**Purpose:**

The network theory of mental disorders offers a new perspective for the understanding of comorbidities, but the research on the comorbidities among depression, anxiety, and post-traumatic stress disorder (PTSD) is still insufficient. The aim of this study was to explore the internal relationship by establishing and analyzing the comorbidity networks, and to provide suggestions for the intervention after traumatic events.

**Methods:**

We utilized data from the second and third wave of the Chengdu Positive Child Development cohort (*N* = 3,189, 47.79% female), we estimated to network models of depression, anxiety and PTSD. To assess difference in global connectivity between the two networks, we conducted invariance test.

**Results:**

K27 (Somatic 10), K37 (Generalized Anxiety 9), K15 (Somatic 5), K33 (Generalized Anxiety 7), K24 (Somatic 9) were the most central nodes in both networks, P13 (Sleep problem) had the highest Bridge Expected Influence value. The structural difference between the two networks was statistically significant (M = 0.229, *p* = 0.010), and the global strength of the network at wave 2 was higher than the network at wave 3 (35.1 vs. 33.9, S = 1.20, *p* = 0.010).

**Conclusion:**

The correlation in symptoms of the three disorders underscores the need for more comprehensive treatment options for intervention after traumatic events. Central and bridge nodes could inform targeted interventions or policy decisions. Anxiety disorders, especially Som and Gen dimensions, should be the focus of intervention. The Arousal dimension in PTSD, especially sleep disorders, may contribute to the comorbidities. In addition, this study highlights the importance of staged post-traumatic interventions.

## 1 Introduction

Anxiety disorder is a global mental health concern. According to The China Mental Health Survey conducted between July 22, 2013, and March 5, 2015, anxiety disorder has the highest incidence of mental illness, with a lifetime weighted incidence of 6.3% (1). Moreover, it frequently co-occurs with other psychological symptoms, particularly depression and post-traumatic stress disorder (PTSD) (2). Adolescents are more susceptible to mental health issues (3). It is estimated that 14% of adolescents (aged 10–19) experienced mental health problems globally in 2019, with depression disorder and anxiety disorder being significant contributors to adolescent illness and disability (4). Depression and anxiety can impact adolescents’ daily lives and, in severe cases, lead to self-harm or suicide (5–7). Additionally, adolescent mental health are linked to negative long-term outcomes in adulthood, such as physical and mental health, social functioning, and educational achievements (8).

The global COVID-19 pandemic and the associated public health measures implemented to slow down the virus spread, has affected adolescents’ activities and may have long-term negative effects on their mental health (9–11). As a novel and unique global stressor, the fear and uncertainty brought by COVID-19 can lead to symptoms of traumatic stress (12). This stress increases the risk of depression and anxiety (13), often coexisting with PTSD (2). Adolescents are more susceptible to the deteriorating effects on their mental health (14). Considering that the high infection rate and long incubation period (15), the potential traumatic impact of the COVID-19 pandemic on adolescents is unlikely to be similar to any disaster outcomes (such as earthquakes or tsunamis) (16). Exploring the coexistence patterns and relationships between depression, anxiety, and PTSD during different stages of the pandemic can inform the development of more effective and context-specific intervention.

Research has shown that PTSD, anxiety, and depression have overlapping risk factors and symptoms. The common trigger factors of PTSD and depression encompass genetic contributors, alongside various environmental influences (11). This phenomenon is caused by the common vulnerability characteristics (17), though evidence remains inconclusive. Anxiety disorder also exhibits significant overlap with PTSD and depression in both triggers and symptoms, including sadness, worry and other emotions, sleep disorders, reduced interest in daily activities, etc. (18, 19). Severe anxiety, depression, and PTSD often present with overlapping symptoms, such as eating disorder, social fear disorder, sleep disorder (20, 21). Adolescents with three conditions all have different degrees of sleep disorders, reduced interest in daily activities and difficulty concentrating. In addition, the three disorders share common risk factors (22), including the family environment (23) and the common vulnerability of individual disease (17).

In previous research on mental disorders, the most popular model is the potential variable model (24). This model shows that the symptoms are caused by unobserved potential variables. The correlation between symptoms can be explained by potential variables. But this may neglect important nuances in how symptoms of each diagnosis affect one another (25). In recent years there has been a shift from a latent-based approach to a network approach, in order to explain the correlation among variables (26). The network structure is an alternative framework for conceptualizing mental disorders, which enables dynamic system modeling programs on the existing links among the three conditions, and emphasis on understanding the strength and nature of associations among symptoms (27, 28). Networks analysis offers powerful empirical tools to visualize symptoms of in large-scale epidemiological studies (29). With this framework, the pathogenesis is tracked according to the symptoms and etiology analysis of existing diseases (30). In addition, contact between diseases enables direct, intuitive, and detailed description of the complex associations between symptoms.

Despite the recognized importance, research investigating the predictors and co-disease relationship between the three disorders is currently limited. Most existing studies are based on cross-sectional data. At the same time, most studies targeted at specific people and populations (31–33), which are less replicable and generalizable. While some have been studies investigating the pairwise relationships between the depression, anxiety, and PTSD two by two in different groups, including randomly selected volunteers (21, 34) and veterans (35), research on young people with a high incidence of the disease is still insufficient (36). Adolescents have a high risk of developing mental disorders, particularly anxiety and depression. Without timely and effective treatment, these conditions can lead to long-term consequences (37). In addition, given China’s unique cultural background, its network structure may be different from that of other countries (38, 39).

To address these research gaps, this study examines the co-occurrence of depression, anxiety, and PTSD using data from a short-term longitudinal cohort of primary school students in Chengdu (*n* = 3,533). We employ network analysis and network comparison to explore their relationships. The network structure facilitates intuitively and efficiently find the relationship between the three disorders. Furthermore, centrality analysis aids in find the internal connection between the pathogenesis and external manifestations of the three diseases and further study prevention or treatment measures. Network comparison is utilized to explore the vertical relationship between networks. It is speculated that there should be a long-term relationship between the pathogenesis of anxiety disorder, depression disorder and PTSD. Nevertheless, this study acknowledges that the mechanisms linking these disorders may change over time.

Thus, the aims of this study are as follows:

- Aim 1: Model the accurate depression-anxiety-PTSD network structure and identify the most central nodes.
- Aim 2: Analyze the bridge nodes among depression, anxiety, and PTSD to identity key symptoms of comorbidities.
- Aim 3: Compare the comorbid networks at wave 2 and wave 3 to test the hypothesis that comorbidity networks change over time and identify effective interventions for post-traumatic psychological symptoms in different stages.

## 2 Materials and methods

### 2.1 Participants and sampling approach

Data used in this study were derived from the Chengdu Positive Child Development (CPCD) study (40). In CPCD study, data were collected three times from five primary and junior high schools (i.e., from grade 1 to grade 9) in Chengdu during 2019 and 2021. The CPCD using multi-stage stratified cluster sampling method to select five primary and middle schools: one downtown, two suburban in the south and two suburban in the north of Chengdu. The first data collection (Wave 1, did not collect PTSD scale) took place at the end of 2019 before the outbreak of the COVID-19, the second (Wave 2) was between June and July 2020 after the resumption of face-to-face schooling, and the third (Wave 3) was in June 2021. The students were invited to respond to a questionnaire in their classrooms during school hours. Considering the loss of follow-up due to higher education, students from grades 2–5 who participated in the second and third data collection were selected as respondents. After excluding invalid data, 3,189 out of 3,533 students were included in the final analysis. The attrition analyses revealed no significant differences between the sample in age and gender. In terms of ethical approval, this project was reviewed and approved by the Medical Ethics Committee of Sichuan university (K2020025). Written informed consent was gained from school principals, students, and parents before the data collection.

### 2.2 Instruments

Taking into account the specific population selected for this study, our measurement tools are primarily targeted at children and have been commonly applied in prior Chinese research, showing adequate psychometric properties.

#### 2.2.1 Depression Scale for Children

Center for Epidemiologic Studies Depression Scale (CES-D) is a 20-item self-report questionnaire for screening depression and assessing the frequency of its symptoms (41). Each of the 20 items describes a feeling or behavior related to different depressive symptoms. The participants needed to select how often they having the feeling or engaging in the behavior in the past week through a scale with four points (“0” = “rarely or none of the time (<1 day),” “1” = “some or little of the time (1–2 days),” “2” = “moderately or much of the time (3–4 days),” and “3” = “most or almost all the time (5–7 days)”). The CES-D have been widely used and validated across different age groups, including adolescents (42, 43). Previous studies support the factorial validity of the Chinese CES-D (44). The scale score was the sum of the four items scores, which measure “positive affect” that were reversely coded. Higher composite score indicates higher depression. Scores over 15 can be indicative of significant levels of depression disorder (45). In the present study, the scale showed adequate internal consistency (see Supplementary Table S1).

#### 2.2.2 Child Anxiety Related Emotional Disorders

Anxiety was examined through the questionnaire named “Screen for Child Anxiety Related Emotional Disorders (SCARED)” (46, 47). The SCARED assesses children’s anxiety disorders, and it includes 41 items under five dimensions: “Panic/Somatic (Som),” “Generalized Anxiety (Gen),” “Separation Anxiety (Sep),” “Social Phobia (Soc),” and “School Phobia (Sch).” Being widely validated in different adolescent populations, the scale demonstrated stable factor structure and good reliability (48). Through a 3-point scale, the participants evaluated each item to indicate the level of experiencing the specified anxiety symptom (“0” = “Never,” “1” = “Sometimes,” and “2” = “Often”). A higher score (i.e., the sum of all item scores) refers to a higher level of anxiety disorder. A total score of ≥25 may indicate the presence of an Anxiety Disorder (46). In the present study, the scale showed adequate internal consistency (see Supplementary Table S1).

#### 2.2.3 The Children’s Impact of Event Scale (13)

Adolescents’ PTSD were assessed using the Children’s Revised Impact of Event Scale (CRIES-13) with reliable psychometric properties (49). This scale was used as an objective assessment instrument to screen for PTSD after different traumas (e.g., hurricane) among Chinese children and adolescents, and it includes 13 items under three dimensions: “Intrusion (Int),” “Avoidance (Avo),” and “Arousal (Aro)” (50, 51). Participants were asked to rate the frequency of occurrence of each symptom using a 4-point scale (0 = “never” 1 = “rarely” 3 = “sometimes” and 5 = “a lot”). A cutoff score of 30 was applied to indicate the prevalence of PTSD (49). In the present study, the scale showed adequate internal consistency (see Supplementary Table S1).

Cronbach’s *α* coefficient was used to assess the internal consistency reliability of the CSE-D, SCARED and CRIES-13 (see Supplementary Table S1) (52).

### 2.3 Study variables

Mental health variables: depression, anxiety, and PTSD were the key outcomes in the current study. Other demographic variables included grade and gender (1 = Male, 2 = Female). Because the study focused on the complex interactions within comorbidity networks, other confounders were not included.

### 2.4 Data analysis

Our study determined the necessary sample size, accounting for the cluster sampling method used. According the meta-analysis, the pooled prevalence (*p*) of depression, anxiety, and PTSD among adolescents in China during the pandemic were 29, 26, and 48%, respectively (53). To achieve a relative error (*ε*) of 10%, we calculated the sample size using the formula described by Huang et al. (1).


$$N=\frac{Z_{1-\frac{\alpha }{2}}^{2}\left(1-p\right)}{p\varepsilon^{2}}$$

The calculated sample size was 1,093 (*α* = 0.05, *p* = 26%). Due to the design of cluster sampling, we estimated the necessary sample size at approximately 2,200 participants using a design effect of 2 to account for the cluster sampling design (the final sample size = the calculated sample size * the design effect). To detect an effect size of 0.2 at a significance level of 0.05 with 0.90 statistical power (*d* = 0.20, *α* = 0.05), a minimum of 265 participants was required [265 was conducted using G*Power software (version 3.1)] (54). Therefore, the sample size of 3,189 participants in this study was sufficient.

All statistical analyses were conducted using R software (version 4.2.3). Data were cleaned and the multiple imputation method is used to impute these missing data via the “mice” package in R (version 4.2.3) and a total of 5 imputed datasets were obtained and analyzed. Conduct descriptive statistics on the data to summarize the demographic information of participants, as well as the average and standard deviation of the project.

Network structure: A comorbidity network model for two rounds of investigation was constructed using the R package networktools (55) and the GLASSO algorithm (56). The introduction of a LASSO penalty factor to regularize the network and avoid identifying spurious correlations (57). Network analysis was selected as it provides a graphical representation of the conditional relationships between symptoms, offering a nuanced perspective on their interconnections beyond traditional statistical approaches such as regression or factor analysis. The network structure centrally reflects the importance of nodes (58). Nodes in the network structure represent symptoms and edges in this model are interpreted as partial correlation coefficients ranging from −1 to 1, reflecting the paired conditional relationship between two nodes and controlling all other nodes in the network. Red edges signify negative associations and green positive associations. The thicker and more saturated the edge, the stronger the relationship between nodes (58). In this study, the communities were defined based on the theoretical constructs underlying the questionnaires, and symptoms with similar meanings (e.g., sleep problems across different scales) were not merged to maintain interpretability.

Centralities analysis: To determine the bridging effect of symptoms in each comorbidity network, we used the centrality index Expected Influence (EI) to quantify the importance of each symptom node in the network model, as it has a strong correlation with the observed node impact (59). The “central symptom” may have a greater impact on other nodes (60). Another centrality index, bridge centrality, is used to investigate the comorbidity across mental disorders (61). Bridge centrality identifies “bridge symptoms” that connect two mental disorders (62). Bridge Expected Influence (bEI) was calculated as a measure of bridge centrality using the function bridge of the R package networktools (version 1.4.0) (55) to evaluate the significance of a node in connecting external symptom dimensions.

Robustness tests: Tests of robustness comprised 2,500 bootstrapped 95% confidence intervals (CIs) for edge weights and the correlation stability coefficient (CS coefficient) for EI and bEI in subset sample dropping bootstrapping tests (63). Less CI overlap indicates more accurate edge weights (57). CS coefficients above 0.25 are acceptable, and above 0.50 are good (63). The R package bootnet (63) was used to conduct robustness tests, using a non-parametric bootstrap program to estimate edge stability.

Network comparisons: The R packet Network Comparison Test (NCT) (64) was used to test if the two networks differed significantly from each other in global structure and global strength. NCT is a permutation-based network comparison test in which the original group members are repeatedly randomly reassigned to new subsamples that maintain the original sample sizes (1,000 times). A comparison of network structures may yield more information.

## 3 Results

### 3.1 Selected characteristics of participants

Of the 3,189 respondents who screened into the depression, anxiety, and PTSD diagnostic modules, 1,524 were female (47.8%). Supplementary Table S1 shows the mean scores, standard deviations for each symptom and dimensions of symptoms on the depression, anxiety, and PTSD, including a correlation matrix of depression, anxiety and PTSD is presented. Additional descriptive statistics are presented in the Supplementary Table S1.

### 3.2 The network structure

**Figure 1:**
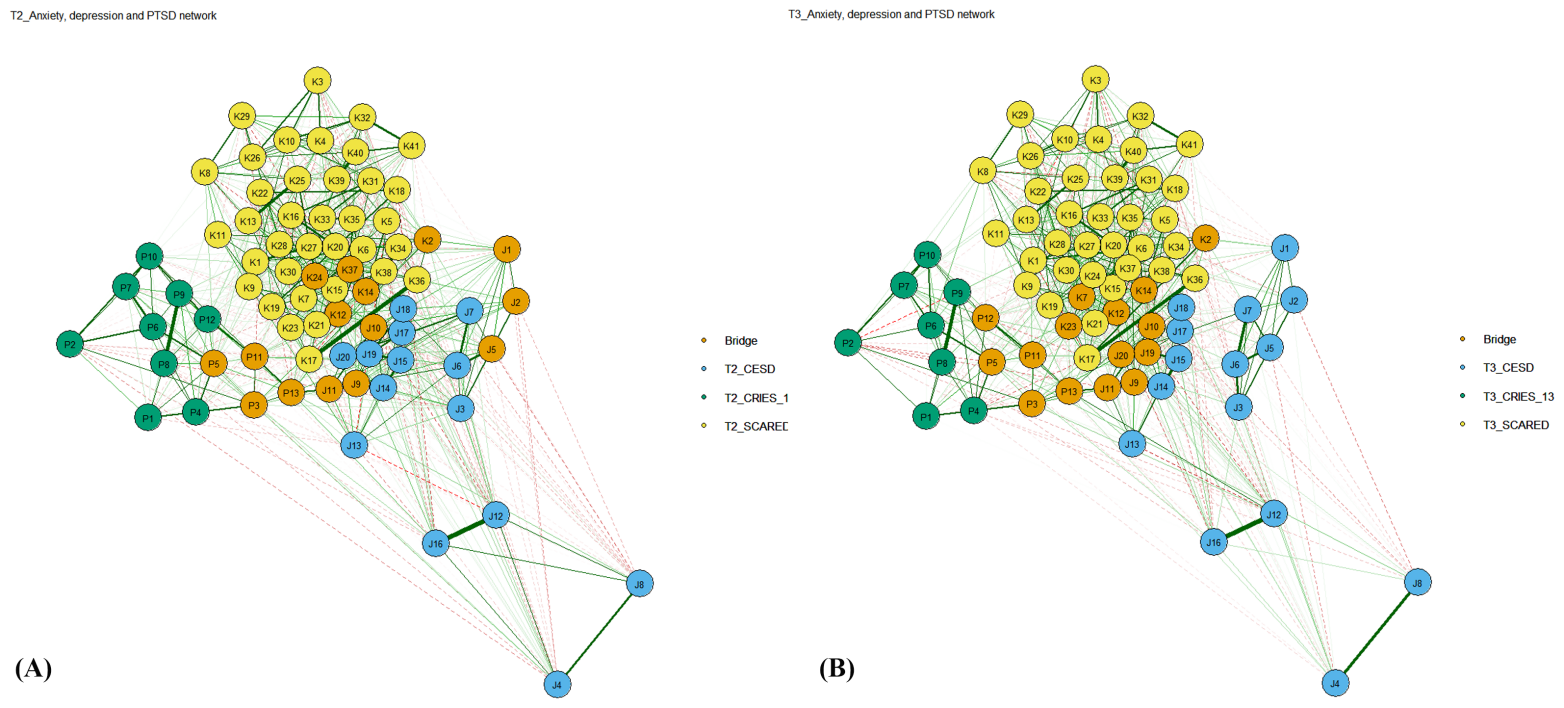
Depression-anxiety-PTSD network in the second and third wave study samples from CPCD. T2: the second wave, T3: the third wave. CESD = Depression SCARED = Anxiety CRIES_13 = PTSD. **(A)** Depression-anxiety-PTSD network in the second wave. Number of nodes: 74. Number of non-zero edges: 439 / 2701. Mean weight: 0.011426. **(B)** Depression-anxiety-PTSD network in the second wave. Number of nodes: 74. Number of non-zero edges: 375 / 2701. Mean weight: 0.011446.

The network constellations of depression, anxiety and PTSD with bridge symptoms are presented in Figure 1. There are no isolated nodes in the network; all symptoms are connected, either directly or indirectly via other symptoms. The positioning of all network nodes is in similar patterns in both two time-points. While most nodes are clustered together in a single cluster, excepting J4 (Good), J8 (Hopeful), J12 (Happy), J16 (Enjoy). The cluster is comprised of three main groups of sparsely connected symptoms, representing depression, anxiety, and PTSD. The strongest edge connections emerged between symptoms P8 (Flashback) and P9 (Upset by reminders), K13 (Sep 3) and K25 (Sep 6), K17 (Soc 3) and K36 (Soc 4), J6 (Depressed) and J7 (Effort), J12 (Happy) and J16 (Enjoy), J4 (Good) and J8 (Hopeful).

Robustness tests were used to assess the stability and accuracy of the two networks (see Supplementary Figures S2, S3). The bootstrapped CIs of edge weights at each wave indicated that the two networks had adequate stability and accuracy.

### 3.3 Centrality analysis

**Figure 2:**
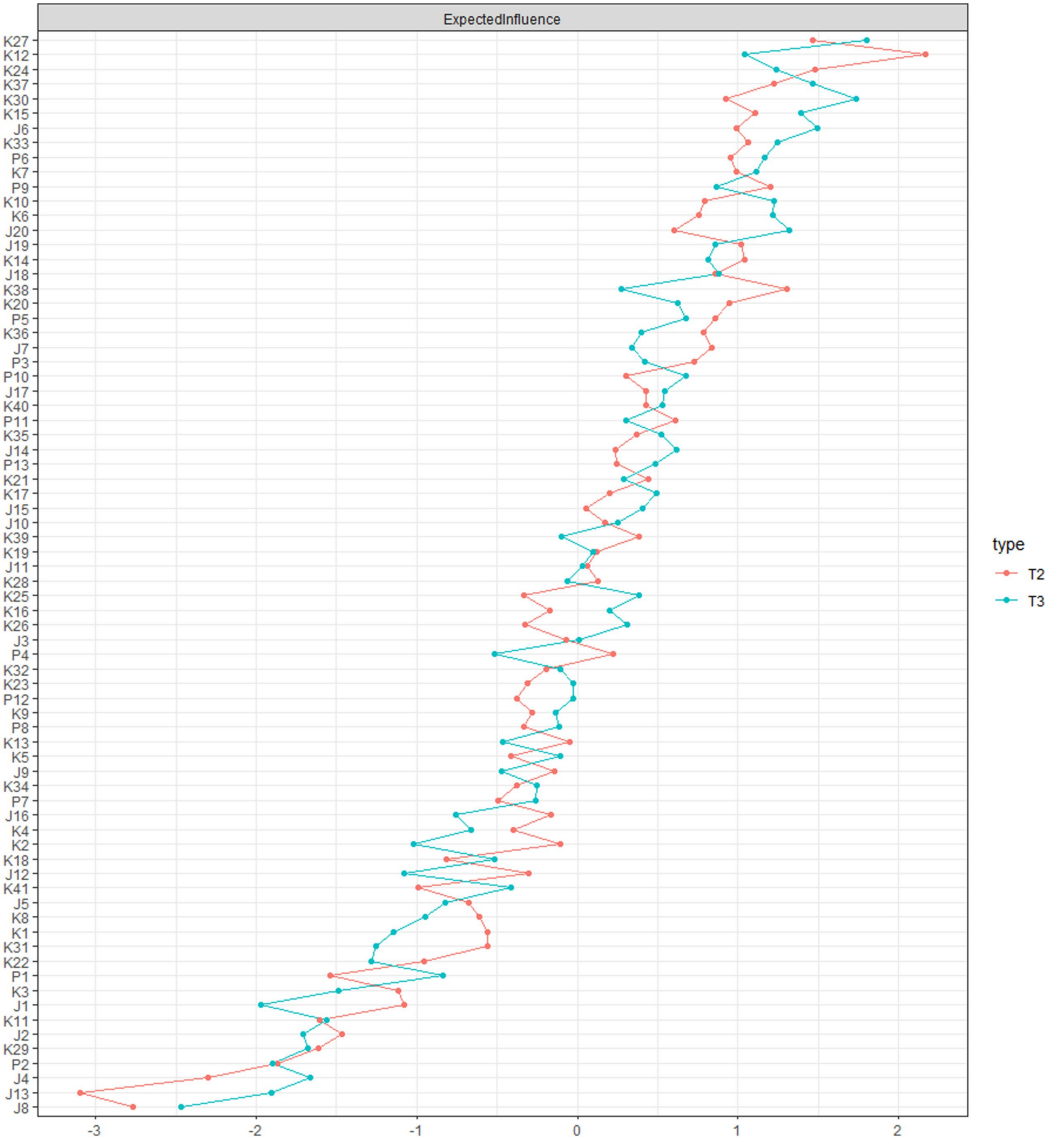
Expected influence estimates for depression-anxiety-PTSD network in the second and third round study samples from CPCD.

Figure 2 shows the results of the centrality analysis (The EI difference tests see Supplementary Figure S4). We found that at the network wave 2, K12 (Som 4), K24 (Som 9), K27 (Som 10), K38 (Som 13), K37 (Gen 9), P9 (Upset by reminders), K15 (Som 5), K33 (Gen 7) had the highest EI, at the network 3, K27 (Som 10), K30 (Som 11), J6 (Depressed), K37 (Gen 9), K15 (Som 5), J20 (Get Going), K33 (Gen 7), K24 (Som 9) had the highest EI. This indicated that these six symptoms were the most central symptoms that have strong direct connections to other neighboring symptoms in the present network from a statistical perspective and thus affect them strongly. Targeting these high-EI symptoms in interventions may help disrupt symptom clustering and reduce overall comorbidity.

**Figure 3:**
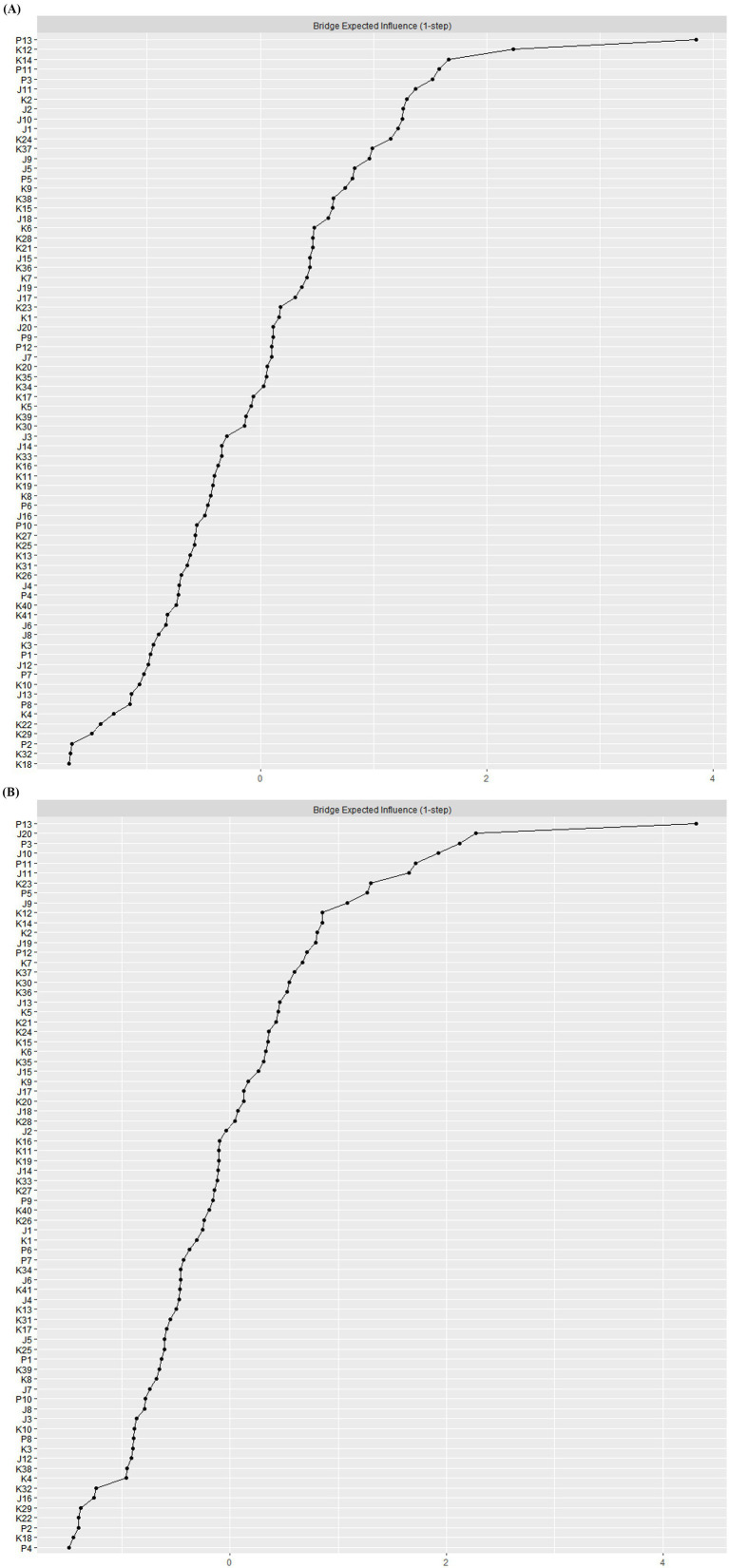
Bridge expected influence estimates for depression-anxiety-PTSD network in the second and third round study samples from CPCD. **(A)** bEI for network in the second wave. **(B)** bEI for network in the third wave.

We used a bridge centrality test to assess the bridge symptoms in both networks (see Figures 1, 3; Supplementary Figure S5). The findings indicated that the two networks shared several bridge symptoms, such as P13 (Sleep problem), P11 (Irritability), P3 (Concentration deficit), J11 (Sleep) are bridge symptoms in depression, anxiety, and PTSD. Given the strong bridging role of sleep-related symptoms, interventions focusing on sleep regulation strategies (e.g., CBT-I, sleep hygiene education) may not only alleviate sleep problems but also weaken the connections between anxiety, depression, and PTSD. We also found unique bridge symptoms in each network. The bridge symptoms in the network at wave 2 included K12 (Som 4), K14 (Gen 3), K2 (Sch 1), J2 (Appetite); J20 (Get Going), J10 (Fearful), J11 (Sleep), K23 (Gen 5) were bridge symptoms in the network at wave 3. These findings suggest that intervention strategies should be time-sensitive, addressing key bridge symptoms as they emerge over time.

The stability of centrality indices was estimated by the correlation stability coefficients (CS-coefficient), which quantifies the maximum percentage of cases that can be dropped to retain stability. A CS-coefficient above 0.5 indicates an acceptable stability of the centrality indices. According to the results (see Supplementary Figure S1), the stability of centrality indices showed a similar pattern with the CS-coefficients above 0.672 for EI and bEI, indicating that these two centrality indices were stable among subset cases at each time-point.

### 3.4 The network comparison

The network invariance test was used to examine the difference in global connectivity between the two networks. The results showed a significant difference between the structures of the two networks (*M* = 0.229, *p* = 0.010), and the global strength of the network at wave 2 was higher than the network at wave 3 (35.1 vs. 33.9, *S* = 1.20, *p* = 0.010).

## 4 Discussion

By establishing comorbidity networks, our study found that anxiety disorders, particularly the Som and Gen dimensions, exhibited higher centrality in the network, indicating their significance for intervention focus. Sleep disorders demonstrated the highest bridge centrality within the network, suggesting potential key nodes for comorbidities. In terms of global connectivity, the comorbidity network during early trauma stages appeared more tightly connected, implying greater severity and increased difficulty in intervention (58). Our findings provide evidence for the comorbidity mechanism between these three psychiatric symptoms after the COVID-19 outbreak and offer insights for effective and targeted interventions for child psychopathy in clinical practices.

Our first aim was to model the accurate depression-anxiety-PTSD network structure and identify the most central nodes. We found that the networks in both time-points the overall network comprised three main groups of sparsely connected symptoms, representing depression, anxiety, and PTSD. In addition, the strongest edge connections occur between different symptoms of the same construct. Because depression, anxiety and PTSD structurally represent three independent disorders, symptoms from the same construct are more strongly connected with each other than with items from other constructs (65). This finding is consistent with previous results (25, 65, 66). Although the entire cluster was divided into three groups, the symptom correlations among these three disorders underscore the need for more comprehensive treatment options following traumatic events (67).

The most central nodes of the two networks are the same, K27 (Som 10), K37 (Gen 9), K15 (Som 5), K33 (Gen 7) and K24 (Som 9), indicating that these nodes are strongly connected to other nodes. It shows that anxiety is the more central symptom in the two comorbidity networks, and the occurrence of anxiety is often accompanied by depression and PTSD. This finding validates research by Astill et al. that anxiety disorders may play a key role in the development and maintenance of depression and PTSD (68, 69). Intervention programs targeting these important nodes should be considered to potentially make comorbid interventions more effective. Notably, these five nodes belong to only two dimensions of anxiety—Som and Gen. This may be because Som and Gen are the two most important and closely related dimensions in childhood anxiety (70), and these two dimensions should be the focus of intervention. Moreover, given that cultural differences play a crucial role in shaping mental structures and behaviors, cultural orientation can influence children’s responses to stress and anxiety (71). In collectivist China, anxiety was more strongly associated with eating behavior than physical activity (72). Therefore, the formulation of intervention policies needs to integrate cultural values and social structures.

Our second aim was to analyze of bridge nodes among depression, anxiety, and PTSD. The results indicate that the comorbidity of depression, anxiety and PTSD may be attributable to their shared bridge symptoms, P13 (Sleep problem), P11 (Irritability), P3 (Concentration deficit), J11 (Sleep) are bridge symptoms common to both networks. In both networks, P13 was the most important bridge node, and P13 (Sleep problem) and J11 (Sleep) reflected the sleep disorder of the children from two perspectives. Consistent with most studies, there was a strong co-occurrence between sleep disorder and psychiatric disorders (73–75). Adequate sleep duration is a cornerstones of physical well-being, which serves as the foundation for coping with stressors such as social isolation and academic challenges (76). While socio-cultural could have an impact on sleep patterns (77). For Chinese children, lack of sleep is often linked to different times of going to bed and fear of sleeping alone (78). Notably, P13 (Sleep problem), P11 (Irritability), and P3 (Irritability) all belong to the Arousal dimension of PTSD. It may be that arousal in the face of stressors potentially stimulates certain areas of the brain (hippocampus), making victims of previous traumatic events more susceptible to current life stresses (79).

Different from the findings of Matthew et al. (44), PTSD did not show heterogeneity in our comorbidity network, possibly because the traumatic event in this study was COVID-19 outbreak, while the Matthew’s study did not target a specific traumatic event. The central nodes and the bridge nodes do not coincide because they represent different meanings in the network (80). Furthermore, the central node or bridge node identified by different studies may be different, and this difference may be caused by the differences in traumatic events, measurement tools and sample types (81). This highlights the importance of comorbidity of specific traumatic events for different populations in the development of personalized intervention programs.

Our third aim was to compare the comorbid networks at wave 2 and wave 3. The findings indicate that the structure of the two networks varies, suggesting that the network structure may change with the time of the traumatic event. This is consistent with our hypothesis. The global strength of the network at wave 2 was higher than the network at wave 3, suggesting that the comorbidity characteristics in children are more obvious in the second round than in the third round after the COVID-19 outbreak. This is consistent with the results of Qi et al.’s study (80) on PTSD and depression comorbidity. PTSD and depression became more independent over time, as negative psychological symptoms gradually separated into different disorders over time. After the peak of the COVID-19 outbreak, lockdown and quarantine measures were gradually lifted in most areas and schools reopened; these shifts toward regular routines may have contributed to more attenuated connections between these nodes within the symptom network following the pandemic peak (82–84). These findings suggest that the development of interventions for post-traumatic psychological symptoms should change over time: in the early stages, interventions should focus on comorbid symptoms, whereas in later stage, individualized treatments may be more effective.

Specifically, there were different central nodes and bridge nodes in the two networks. For example, as for the central nodes, P9 (Upset by reminders) appeared in the second round of the network, while J6 (Depressed) appeared in the third round of the network. It may be because the fact that the second round of the survey was at the beginning of the global pandemic of COVID-19, when thinking about the outbreak triggered additional psychological symptoms. This finding aligns with “reliving trauma” being an important symptom in the symptoms of PTSD (69). By the time of the third survey, people had become accustomed to the COVID-19, and thinking about the COVID-19 no longer played a significant role in developing other psychological symptoms. On the contrary, prolonged depression due to the COVID-19 pandemic may play a more important role in the occurrence of other psychological symptoms (85). This is consistent with the comparison of the global strength of the two waves of networks. Regarding bridge nodes, K12 (Som 4) was seen in the second round of the network, while J20 (Get Going) was seen in the third round of the network. This may be because the stress associated with COVID-19 may bring prolonged uncertainty or worry (86), leading to further psychological symptoms, such as feeling low mood, rather than somatic symptoms (87).

Furthermore, stress related to COVID-19 may raise uncertainty and chronic worry, contributing to further psychological symptoms such as fear, restlessness, and irritability rather than somatic symptoms. Multiple studies have also confirmed the delayed impact of the COVID-19 outbreak on mental health, with different symptoms developing in different developmental sequential (88), underscoring the importance of expanding longitudinal studies (89, 90). These findings may enhance our understanding of the comorbidity among depression, anxiety, and PTSD across trauma time, and highlight the need to consider trauma time as a factor in research on comorbidity and clinical practice.

## 5 Strengths and limitations

Our study holds both theoretical and practical contributions. Firstly, this study employs network analysis to investigate symptom associations and identify key nodes, providing a fresh perspective on the comorbidity structure of depression, anxiety, and. Secondly, it offers a comprehensive evaluation of the comorbidity network for adolescent psychiatric symptoms, focusing on depression, anxiety, and PTSD. Additionally, we conduct assessments at different stages of the COVID-19 pandemic to explore changes in trauma-related network structures over time. By doing so, we address limitations inherent in cross-sectional studies, and offer evidence supporting comprehensive long-term intervention strategies. These findings may help policy makers in their efforts to develop preparedness plans for future unprecedented events and guide mental health professionals and school personnel to support children now and in the future.

Some limitations of this study also need to be acknowledged. First, while the aim of this study was to examine the comorbidity of depression, anxiety, and PTSD, all the participants were nonclinical samples relying solely on self-reported psychometric data for symptom assessment. As a result, participants may exhibit subliminal symptoms which may not fully reflect the severity and complexity observed in clinical cases. This limitation makes it challenging to assess comorbidity in depth and restricts the generalizability of our findings to clinical populations. Future research should validate these findings in diagnosed clinical samples to enhance applicability. In addition, self-reported bias also affected the accuracy of the study results. Second, our study focused on COVID-19 as a single trauma type and was limited to Chengdu, which limits the exploration of different trauma types and different regions. Third, we aimed to examine the symptom structure of diagnostic categories and so included all symptoms. It is possible that some of the nodes in the network represent the same semantic cluster (e.g., J11: Sleep and P13: Sleep problem), rather than the interaction of otherwise independent constructs. Finally, all analyses were highly exploratory, and as such should be interpreted as hypothesis generating rather than confirming effects.

Further studies should examine whether similar patterns hold in clinical settings, where symptom severity, comorbidities, and treatment histories may introduce additional complexities. Various types (such as car accidents or sexual assaults) and different regions of traumas can also be expanded to broaden the application of comorbidity networks across diverse traumatic experiences and across different regions. Additionally, longitudinal studies are warranted to explore long-term changes in comorbid structures following trauma and furnish stronger evidence for comprehensive long-term intervention strategies.

## 6 Conclusion

In conclusion, our findings underscore the importance of prioritizing anxiety disorders when addressing comorbidities associated with depression, anxiety, and PTSD, while also targeting sleep disorders to mitigate their co-occurrence. In addition, post-traumatic intervention strategies need to consider stage-specific approaches as well as encompassing both comprehensive and targeted measures. Future studies should emphasize on long-term monitoring of post-traumatic stress symptoms to provide further evidence. We also call for more future research into other types of trauma and health emergencies.

## Data Availability

The original contributions presented in the study are included in the article/Supplementary material, further inquiries can be directed to the corresponding author.
