# Supplementary material for: Longitudinal network analysis of depression, anxiety, and post-traumatic stress disorder comorbidities among adolescents in regional China
Source: Front Public Health. 2025 Mar 17;13:1522877. doi: 10.3389/fpubh.2025.1522877 (PMC11955477; doi:10.3389/fpubh.2025.1522877)
Supplement: Supplementary file 1 [file Data_Sheet_1.docx]

Supplementary Material

# Page 2. Table S1 Descriptions, reliability, and correlations

# Page 3-4. Table S2 Full item text, item shorts, and descriptive statistics

# Page 5. Figure S1 Subset samples-dropping bootstrapping tests

# Page 6. Figure S2 Bootstrapped confidence intervals (CIs) of the edge weights

# Page 7. Figure S3 Edge weight difference tests for networks

# Page 8. Figure S4 The expectedInfluence difference tests

Page 9. Figure S5 Bridge expectedInfluence difference tests

**Supplementary Table 1.** Descriptions, reliability, and correlations (N = 3, 189).

|  | Variable | Descriptions | | |  | Reliability |  | Correlations | | | | | | | | | | | | | | | | | | | | | | | |
| --- | --- | --- | --- | --- | --- | --- | --- | --- | --- | --- | --- | --- | --- | --- | --- | --- | --- | --- | --- | --- | --- | --- | --- | --- | --- | --- | --- | --- | --- | --- | --- |
|  |  | Range | Mean | SD |  | Cronbach’s Alpha |  | 1 | 2 | 3 | 4 | 5 | 6 | 7 | 8 | 9 | 10 | 11 | 12 | 13 | 14 | 15 | 16 | 17 | 18 | 19 | 20 | 21 | 22 | 23 | 24 |
| 1 | Grade | 2-5 | 3.700 | 0.955 |  |  |  | - |  |  |  |  |  |  |  |  |  |  |  |  |  |  |  |  |  |  |  |  |  |  |  |
| 2 | Gender^a^ | 1-2 | - | - |  |  |  | -0.001 | - |  |  |  |  |  |  |  |  |  |  |  |  |  |  |  |  |  |  |  |  |  |  |
| 3 | **T2_Depression** | 60 | 13.665 | 10.281 |  | 0.876 |  | -0.024 | -0.025 | - |  |  |  |  |  |  |  |  |  |  |  |  |  |  |  |  |  |  |  |  |  |
| 4 | **T2_Anxiety** | 82 | 14.870 | 14.806 |  | 0.949 |  | 0.052** | 0.053** | 0.604** | - |  |  |  |  |  |  |  |  |  |  |  |  |  |  |  |  |  |  |  |  |
| 5 | T2_Som | 26 | 3.890 | 5.069 |  | 0.894 |  | 0.021 | 0.012 | 0.614** | 0.920** | - |  |  |  |  |  |  |  |  |  |  |  |  |  |  |  |  |  |  |  |
| 6 | T2_Gen | 18 | 2.966 | 3.893 |  | 0.873 |  | 0.066** | 0.042* | 0.640** | 0.917** | 0.826** | - |  |  |  |  |  |  |  |  |  |  |  |  |  |  |  |  |  |  |
| 7 | T2_Sep | 16 | 3.404 | 3.449 |  | 0.779 |  | -0.008 | 0.067** | 0.471** | 0.867** | 0.727** | 0.746** | - |  |  |  |  |  |  |  |  |  |  |  |  |  |  |  |  |  |
| 8 | T2_Soc | 14 | 3.687 | 3.459 |  | 0.815 |  | 0.108** | 0.101** | 0.293** | 0.760** | 0.567** | 0.598** | 0.597** | - |  |  |  |  |  |  |  |  |  |  |  |  |  |  |  |  |
| 9 | T2_Sch | 8 | 0.921 | 1.421 |  | 0.667 |  | 0.045* | -0.013 | 0.495** | 0.671** | 0.614** | 0.599** | 0.513** | 0.376** | - |  |  |  |  |  |  |  |  |  |  |  |  |  |  |  |
| 10 | **T2_PTSD** | 65 | 16.018 | 15.403 |  | 0.892 |  | -0.017 | -0.036* | 0.307** | 0.427** | 0.406** | 0.405** | 0.363** | 0.300** | 0.283** | - |  |  |  |  |  |  |  |  |  |  |  |  |  |  |
| 11 | T2_Int | 20 | 5.563 | 5.816 |  | 0.792 |  | 0.010 | -0.027 | 0.221** | 0.337** | 0.309** | 0.325** | 0.292** | 0.249** | 0.205** | 0.875** | - |  |  |  |  |  |  |  |  |  |  |  |  |  |
| 12 | T2_Avo | 20 | 5.520 | 5.954 |  | 0.782 |  | -0.043* | -0.044* | 0.164** | 0.299** | 0.281** | 0.270** | 0.266** | 0.223** | 0.183** | 0.861** | 0.619** | - |  |  |  |  |  |  |  |  |  |  |  |  |
| 13 | T2_Aro | 25 | 4.936 | 5.897 |  | 0.761 |  | -0.011 | -0.023 | 0.417** | 0.481** | 0.471** | 0.465** | 0.392** | 0.311** | 0.352** | 0.880** | 0.673** | 0.629** | - |  |  |  |  |  |  |  |  |  |  |  |
| 14 | **T3_Depression** | 60 | 12.965 | 8.505 |  | 0.821 |  | 0.024 | 0.010 | 0.337** | 0.239** | 0.242** | 0.257** | 0.173** | 0.115** | 0.219** | 0.122** | 0.098** | 0.057** | 0.165** | - |  |  |  |  |  |  |  |  |  |  |
| 15 | **T3_Anxiety** | 82 | 15.310 | 14.166 |  | 0.944 |  | 0.045* | 0.035* | 0.128** | 0.170** | 0.165** | 0.163** | 0.135** | 0.114** | 0.131** | 0.076** | 0.077** | 0.046** | 0.075** | 0.549** | - |  |  |  |  |  |  |  |  |  |
| 16 | T3_Som | 26 | 3.922 | 4.956 |  | 0.889 |  | 0.000 | 0.007 | 0.112** | 0.149** | 0.164** | 0.135** | 0.114** | 0.080** | 0.120** | 0.061** | 0.065** | 0.035 | 0.061** | 0.522** | 0.907** | - |  |  |  |  |  |  |  |  |
| 17 | T3_Gen | 18 | 3.138 | 3.858 |  | 0.866 |  | 0.076** | 0.039* | 0.132** | 0.159** | 0.149** | 0.168** | 0.121** | 0.103** | 0.123** | 0.082** | 0.087** | 0.047** | 0.082** | 0.578** | 0.907** | 0.784** | - |  |  |  |  |  |  |  |
| 18 | T3_Sep | 16 | 3.285 | 3.183 |  | 0.751 |  | -0.053** | 0.040* | 0.102** | 0.145** | 0.143** | 0.135** | 0.147** | 0.071** | 0.105** | 0.070** | 0.067** | 0.041* | 0.075** | 0.411** | 0.834** | 0.689** | 0.695** | - |  |  |  |  |  |  |
| 19 | T3_Soc | 14 | 3.919 | 3.390 |  | 0.812 |  | 0.132** | 0.052** | 0.069** | 0.116** | 0.088** | 0.106** | 0.077** | 0.146** | 0.061** | 0.044* | 0.042* | 0.038* | 0.035* | 0.306** | 0.759** | 0.545** | 0.617** | 0.540** | - |  |  |  |  |  |
| 20 | T3_Sch | 8 | 1.046 | 1.420 |  | 0.624 |  | 0.049** | 0.004 | 0.131** | 0.141** | 0.138** | 0.141** | 0.101** | 0.072** | 0.172** | 0.058** | 0.055** | 0.033 | 0.064** | 0.436** | 0.664** | 0.586** | 0.567** | 0.492** | 0.392** | - |  |  |  |  |
| 21 | **T3_PTSD** | 65 | 15.013 | 13.720 |  | 0.872 |  | -0.029 | 0.006 | 0.053** | 0.057** | 0.068** | 0.044* | 0.043* | 0.029 | 0.055** | 0.053** | 0.042* | 0.048** | 0.050** | 0.302** | 0.443** | 0.413** | 0.418** | 0.375** | 0.299** | 0.284** | - |  |  |  |
| 22 | T3_Int | 20 | 5.307 | 5.448 |  | 0.795 |  | 0.023 | 0.005 | 0.027 | 0.035* | 0.042* | 0.027 | 0.027 | 0.017 | 0.033 | 0.037* | 0.048** | 0.020 | 0.031 | 0.191** | 0.310** | 0.290** | 0.299** | 0.271** | 0.205** | 0.172** | 0.847** | - |  |  |
| 23 | T3_Avo | 20 | 5.121 | 5.631 |  | 0.775 |  | -0.117** | -0.006 | 0.008 | 0.023 | 0.033 | 0.008 | 0.027 | 0.012 | 0.005 | 0.053** | 0.021 | 0.074** | 0.043* | 0.135** | 0.282** | 0.260** | 0.246** | 0.262** | 0.200** | 0.167** | 0.832** | 0.541** | - |  |
| 24 | T3_Aro | 25 | 4.585 | 5.263 |  | 0.731 |  | 0.025 | 0.016 | 0.102** | 0.088** | 0.099** | 0.079** | 0.056** | 0.045* | 0.104** | 0.044* | 0.039* | 0.025 | 0.052** | 0.446** | 0.531** | 0.497** | 0.518** | 0.417** | 0.352** | 0.384** | 0.840** | 0.593** | 0.539** | - |
| **P*＜0.05, ** *P*＜0.01.  SD = standard deviation.  Som=Somatic, Gen=Generalized anxiety, Sep=Separation anxiety, Soc=Social phobia, Sch=School phobia, Int=Intrusion, Avo=Avoidance, Aro=Arousal.  ^a^ Male =1, Female =2. | | | | | | | | | | | | | | | | | | | | | | | | | | | | | | | |

**Supplementary Table 2.** Full item text, item shorts, and descriptive statistics of the CES-D, SCARED and CRIES_13 (N = 3, 189)

| **Scale/Item** | | **Item short** |  | **T2** | |  | **T3** | |
| --- | --- | --- | --- | --- | --- | --- | --- | --- |
|  |  |  |  | **Mean** | **SD** |  | **Mean** | **SD** |
| Depression(CES-D) | | |  |  |  |  |  |  |
| J1. | I was bothered by things that usually don't bother me. | Bothered |  | 0.42 | 0.76 |  | 0.45 | 0.75 |
| J2. | I did not feel like eating; I wasn't very hungry. | Appetite |  | 0.53 | 0.85 |  | 0.58 | 0.83 |
| J3. | I wasn't able to feel happy, even when my family or friends tried to help me feel better. | Blues |  | 0.40 | 0.79 |  | 0.39 | 0.74 |
| J4. | I felt like I was just as good as other kids. | Good |  | 1.79 | 1.27 |  | 1.67 | 1.26 |
| J5. | I felt like I couldn't pay attention to what I was doing this week. | Mind |  | 0.40 | 0.75 |  | 0.37 | 0.69 |
| J6. | I felt down and unhappy this week. | Depressed |  | 0.43 | 0.83 |  | 0.41 | 0.76 |
| J7. | I felt like I was too tired to do things this past week. | Effort |  | 0.43 | 0.81 |  | 0.42 | 0.76 |
| J8. | I felt like something good was going to happen. | Hopeful |  | 2.01 | 1.13 |  | 1.99 | 1.10 |
| J9. | I felt like things I did before didn't work out right. | Failure |  | 0.49 | 0.85 |  | 0.46 | 0.80 |
| J10. | I felt scared this week. | Fearful |  | 0.37 | 0.80 |  | 0.30 | 0.70 |
| J11. | I didn't sleep as well as I usually sleep this week. | Sleep |  | 0.49 | 0.91 |  | 0.43 | 0.84 |
| J12. | I was happy this week. | Happy |  | 1.34 | 1.31 |  | 1.26 | 1.25 |
| J13. | I was more quiet than usual this week. | Talk |  | 0.60 | 0.96 |  | 0.48 | 0.84 |
| J14. | I felt lonely, like I didn't have any friends. | Lonely |  | 0.46 | 0.88 |  | 0.42 | 0.85 |
| J15. | I felt like kids I knew were not friendly or that they didn't want to be with me. | Unfriendly |  | 0.44 | 0.87 |  | 0.40 | 0.80 |
| J16. | I had a good time this week. | Enjoy |  | 1.38 | 1.32 |  | 1.34 | 1.30 |
| J17. | I felt like crying this week. | Cry |  | 0.42 | 0.87 |  | 0.41 | 0.83 |
| J18. | I felt sad. | Sad |  | 0.45 | 0.87 |  | 0.44 | 0.83 |
| J19. | I felt people didn't like me this week | Dislike |  | 0.41 | 0.85 |  | 0.37 | 0.78 |
| J20. | It was hard to get started doing things this week. | Get Going |  | 0.41 | 0.83 |  | 0.35 | 0.75 |
| Anxiety(SCARED) | | |  |  |  |  |  |  |
| K1. | When I feel frightened, it is hard for me to breathe. | Som 1 |  | 0.24 | 0.52 |  | 0.29 | 0.56 |
| K2. | I get headaches when I am at school. | Sch 1 |  | 0.30 | 0.55 |  | 0.37 | 0.59 |
| K3. | I don’t like to be with people I don’t know well. | Soc 1 |  | 0.73 | 0.82 |  | 0.87 | 0.79 |
| K4. | I get scared if I sleep away from home. | Sep 1 |  | 0.51 | 0.73 |  | 0.55 | 0.74 |
| K5. | I worry about other people liking me. | Gen 1 |  | 0.39 | 0.67 |  | 0.45 | 0.70 |
| K6. | When I get frightened, I feel like passing out. | Som 2 |  | 0.24 | 0.56 |  | 0.25 | 0.58 |
| K7. | I am nervous. | Gen 2 |  | 0.31 | 0.60 |  | 0.29 | 0.57 |
| K8. | I follow my mother or father wherever they go. | Sep 2 |  | 0.41 | 0.68 |  | 0.33 | 0.61 |
| K9. | People tell me that I look nervous. | Som 3 |  | 0.25 | 0.53 |  | 0.23 | 0.50 |
| K10. | I feel nervous with people I don’t know well. | Soc 2 |  | 0.59 | 0.75 |  | 0.59 | 0.73 |
| K11. | I get stomachaches at school. | Sch 2 |  | 0.34 | 0.59 |  | 0.39 | 0.60 |
| K12. | When I get frightened, I feel like I am going crazy. | Som 4 |  | 0.21 | 0.53 |  | 0.20 | 0.50 |
| K13. | I worry about sleeping alone. | Sep 3 |  | 0.30 | 0.62 |  | 0.24 | 0.55 |
| K14. | I worry about being as good as other kids. | Gen 3 |  | 0.36 | 0.65 |  | 0.38 | 0.65 |
| K15. | When I get frightened, I feel like things are not real. | Som 5 |  | 0.33 | 0.63 |  | 0.32 | 0.61 |
| K16. | I have nightmares about something bad happening to my parents. | Sep 4 |  | 0.30 | 0.59 |  | 0.31 | 0.58 |
| K17. | I worry about going to school. | Soc 3 |  | 0.15 | 0.44 |  | 0.16 | 0.45 |
| K18. | When I get frightened, my heart beats fast. | Som 6 |  | 0.64 | 0.77 |  | 0.68 | 0.78 |
| K19. | I get shaky. | Som 7 |  | 0.29 | 0.59 |  | 0.28 | 0.57 |
| K20. | I have nightmares about something bad happening to me. | Sep 5 |  | 0.40 | 0.66 |  | 0.41 | 0.65 |
| K21. | I worry about things working out for me. | Gen 4 |  | 0.25 | 0.54 |  | 0.25 | 0.54 |
| K22. | When I get frightened, I sweat a lot. | Som 8 |  | 0.47 | 0.69 |  | 0.50 | 0.70 |
| K23. | I am a worrier. | Gen 5 |  | 0.30 | 0.58 |  | 0.29 | 0.57 |
| K24. | I get really frightened for no reason at all. | Som 9 |  | 0.28 | 0.59 |  | 0.26 | 0.56 |
| K25. | I am afraid to be alone in the house. | Sep 6 |  | 0.40 | 0.68 |  | 0.33 | 0.61 |
| K26. | It is hard for me to talk with people I don’t know well. | Soc 3 |  | 0.48 | 0.68 |  | 0.39 | 0.63 |
| K27. | When I get frightened, I feel like I am choking. | Som 10 |  | 0.18 | 0.48 |  | 0.18 | 0.49 |
| K28. | People tell me that I worry too much. | Gen 6 |  | 0.25 | 0.55 |  | 0.24 | 0.53 |
| K29. | I don’t like to be away from my family. | Sep 7 |  | 0.53 | 0.77 |  | 0.47 | 0.74 |
| K30. | I am afraid of having anxiety (or panic) attacks. | Som 11 |  | 0.38 | 0.67 |  | 0.37 | 0.66 |
| K31. | I worry that something bad might happen to my parents. | Sep 8 |  | 0.55 | 0.76 |  | 0.65 | 0.76 |
| K32. | I feel shy with people I don’t know well. | Soc 4 |  | 0.53 | 0.72 |  | 0.60 | 0.72 |
| K33. | I worry about what is going to happen in the future. | Gen 7 |  | 0.42 | 0.69 |  | 0.47 | 0.70 |
| K34. | When I get frightened, I feel like throwing up. | Som 12 |  | 0.19 | 0.49 |  | 0.18 | 0.48 |
| K35. | I worry about how well I do things. | Gen 8 |  | 0.44 | 0.68 |  | 0.50 | 0.70 |
| K36. | I am scared to go to school. | Soc 4 |  | 0.13 | 0.41 |  | 0.13 | 0.41 |
| K37. | I worry about things that have already happened. | Gen 9 |  | 0.26 | 0.56 |  | 0.26 | 0.56 |
| K38. | When I get frightened, I feel dizzy. | Som 13 |  | 0.19 | 0.50 |  | 0.18 | 0.49 |
| K39. | I feel nervous when I am with other children or adults and I have to do something while they watch me (for example: read aloud, speak, play a game, play a sport). | Soc 5 |  | 0.42 | 0.67 |  | 0.47 | 0.68 |
| K40. | I feel nervous when I am going to parties, dances, or any place where there will be people that I don’t know well. | Soc 6 |  | 0.49 | 0.71 |  | 0.53 | 0.71 |
| K41. | I am shy. | Soc 7 |  | 0.44 | 0.67 |  | 0.47 | 0.67 |
| PTSD(CRIES_13) | | |  |  |  |  |  |  |
| P1. | Did you think about it when you didn't mean to? | Intrusive memories |  | 1.29 | 1.81 |  | 1.21 | 1.67 |
| P2. | Did you try to remove it from your memory? | Trauma-related amnesia |  | 1.67 | 2.09 |  | 1.54 | 1.99 |
| P3. | Did you have difficulties paying attention or concentrating? | Concentration deficit |  | 0.84 | 1.53 |  | 0.77 | 1.37 |
| P4. | Did you have waves of strong feeling about it? | Psychological cue reactivity |  | 1.37 | 1.87 |  | 1.40 | 1.79 |
| P5. | Did you startle more easily or feel more nervous than you did before it happened? | Exaggerated startle |  | 1.07 | 1.72 |  | 0.91 | 1.52 |
| P6. | Did you stay away from reminders of it? | Avoiding situations/places |  | 1.21 | 1.80 |  | 1.10 | 1.70 |
| P7. | Did you try not to talk about it? | Avoiding talking about trauma |  | 1.31 | 1.87 |  | 1.27 | 1.80 |
| P8. | Did pictures about it pop into your mind? | Flashback |  | 1.57 | 1.92 |  | 1.43 | 1.78 |
| P9. | Did other things keep making you think about it? | Upset by reminders |  | 1.34 | 1.81 |  | 1.27 | 1.67 |
| P10. | Did you try not to think about it? | Avoiding thoughts |  | 1.33 | 1.89 |  | 1.21 | 1.78 |
| P11. | Did you get easily irritable? | Irritability |  | 1.04 | 1.67 |  | 1.04 | 1.58 |
| P12. | Were you more alert and watchful even when there was no obvious need to be? | Hypervigilance |  | 1.39 | 1.91 |  | 1.26 | 1.77 |
| P13. | Did you have sleep problems? | Sleep problem |  | 0.59 | 1.38 |  | 0.61 | 1.29 |


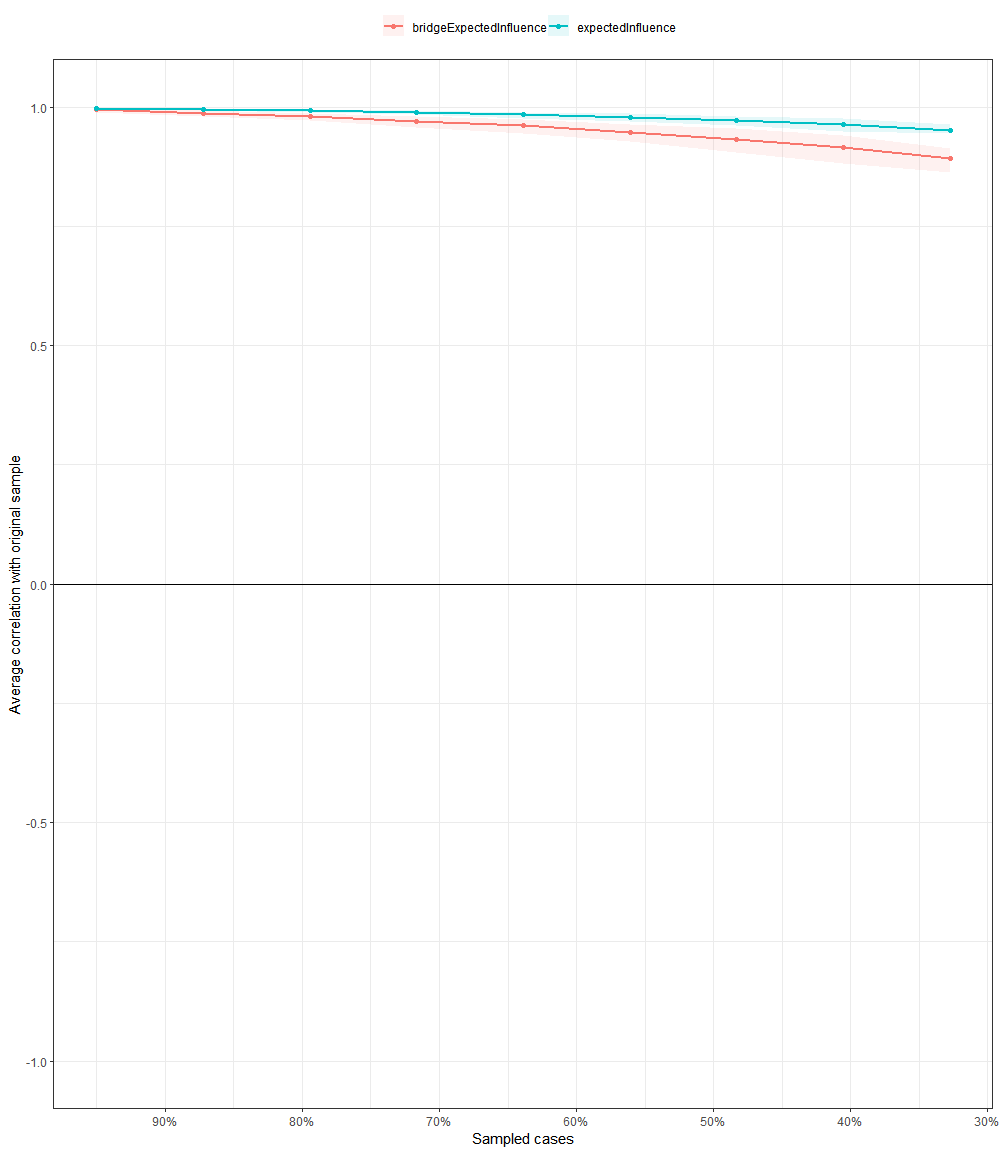


a.Wave 2


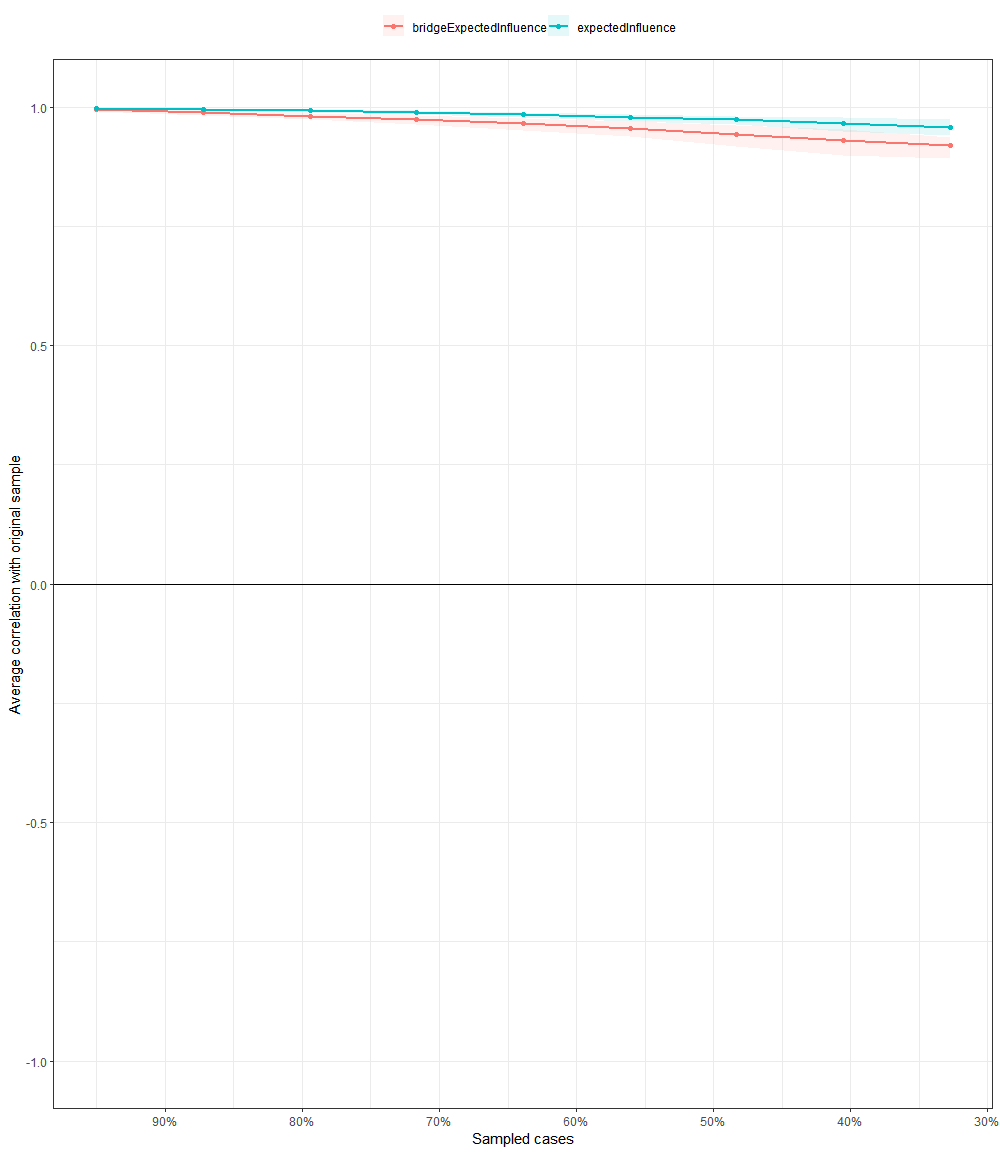


b.Wave 3

**Supplementary Figure 1.** Subset samples-dropping bootstrapping tests for the depression-anxiety-PTSD network in children after COVID-19 in expectedInfluence and bridge expectedInfluence of Wave 2 and Wave 3, respectively.


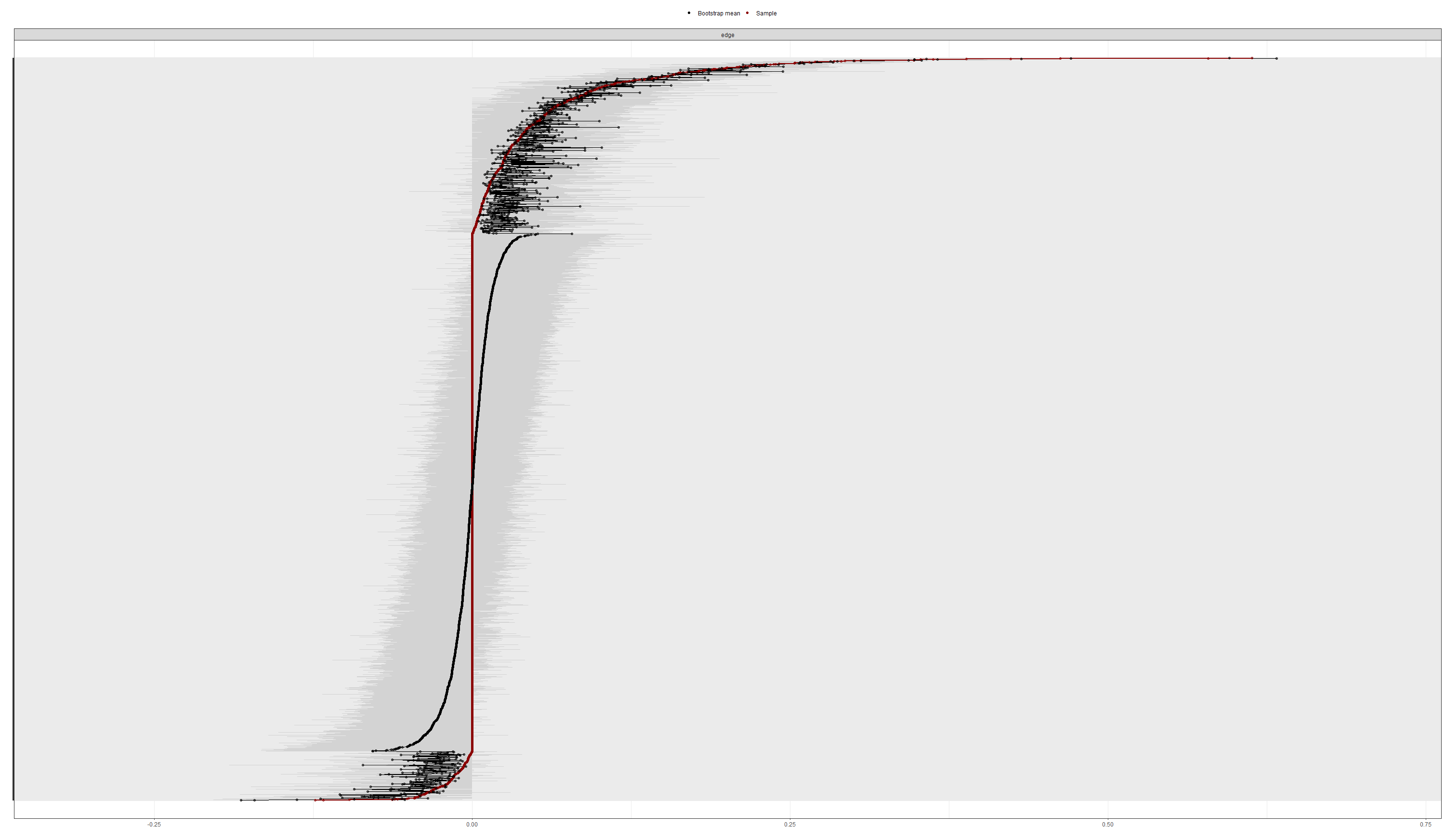


a.Wave 2


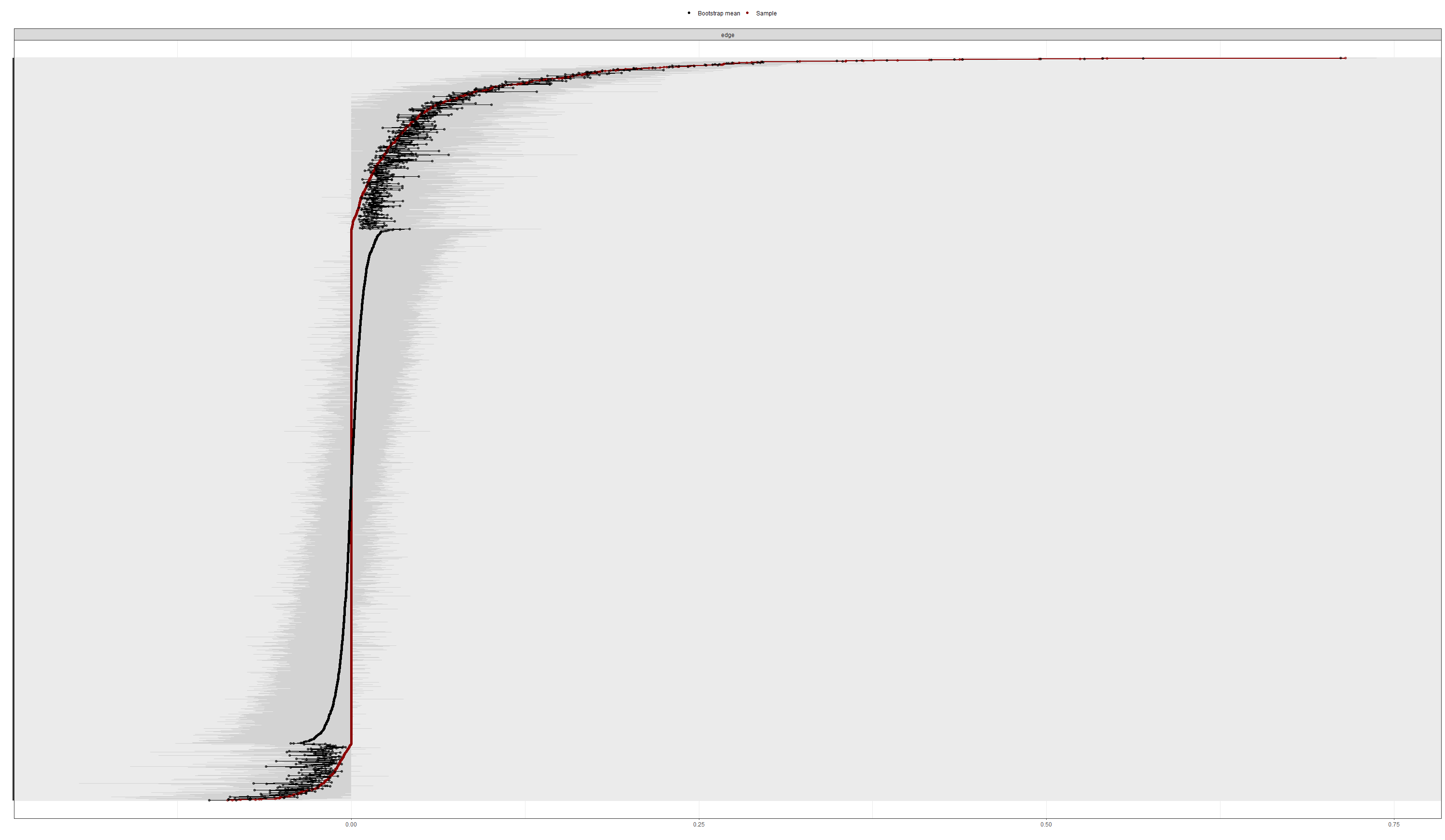


b.Wave 3

**Supplementary Figure 2.** Bootstrapped confidence intervals (CIs) of the edge weights in the depression-anxiety-PTSD network in children after COVID-19 of Wave 2 and Wave 3, respectively.

*Note*:The red line indicates the edge weight values and the gray area the 95% CIs.


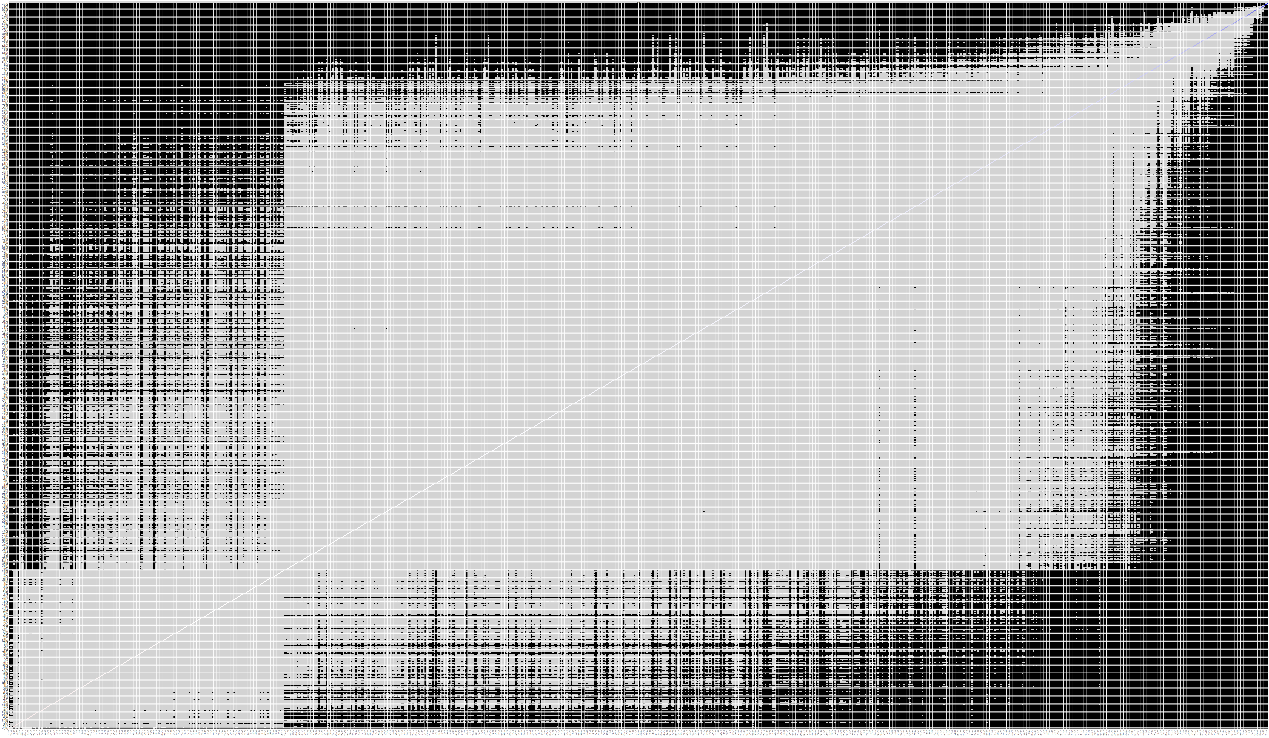


a.Wave 2


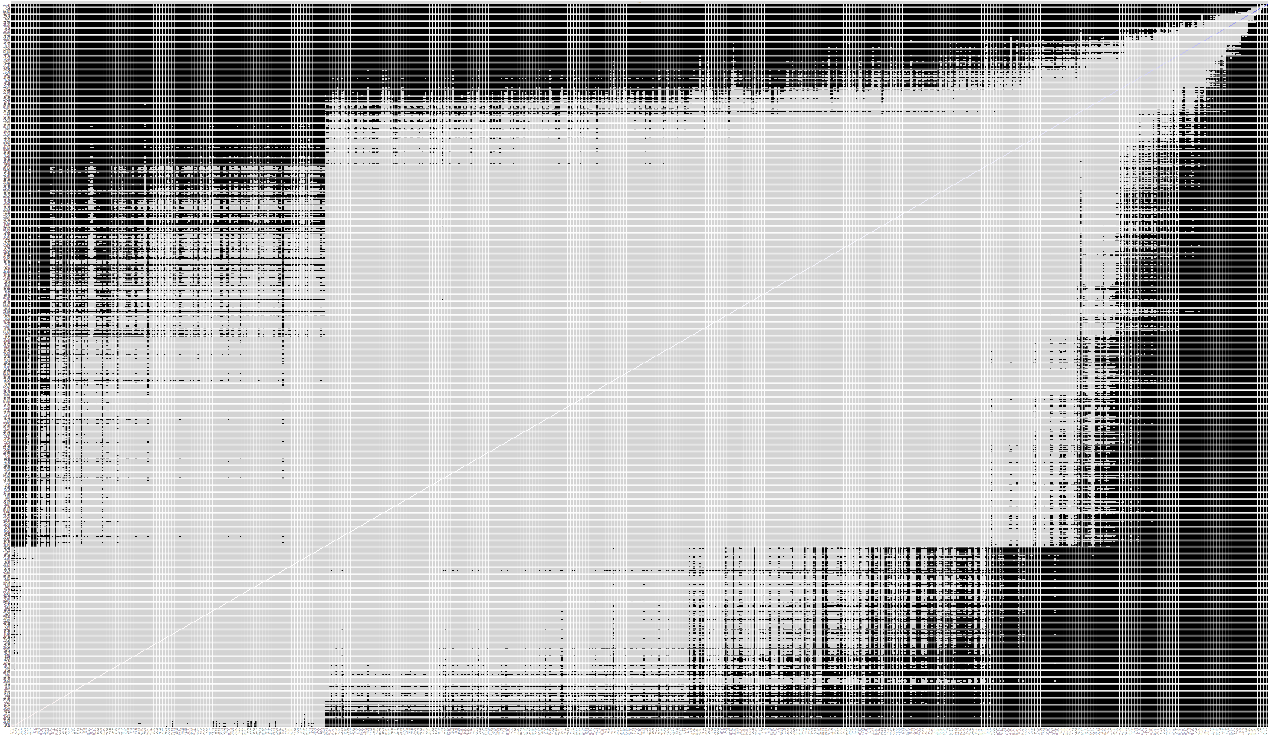


b.Wave 3

**Supplementary Figure 3.** Edge weight difference tests for networks of the depression-anxiety-PTSD network in children after COVID-19 of Wave 2 and Wave 3, respectively.

*Note*: Black box represents an edge that differs significantly (α = 0.05) from another one and grey boxes signify no significant difference. Expected significance level given number of bootstrap samples is approximately: 0.05.


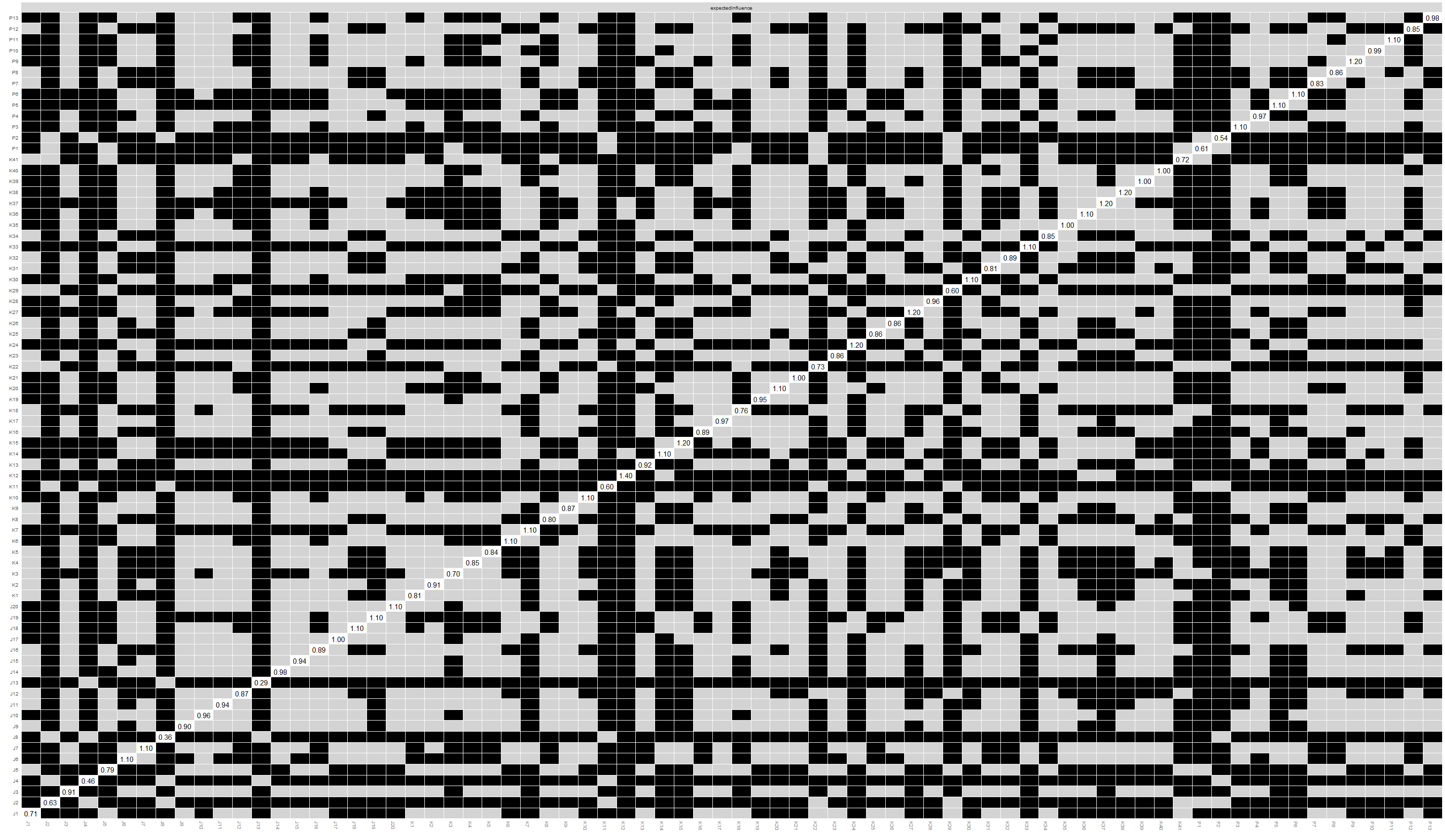


a.Wave 2


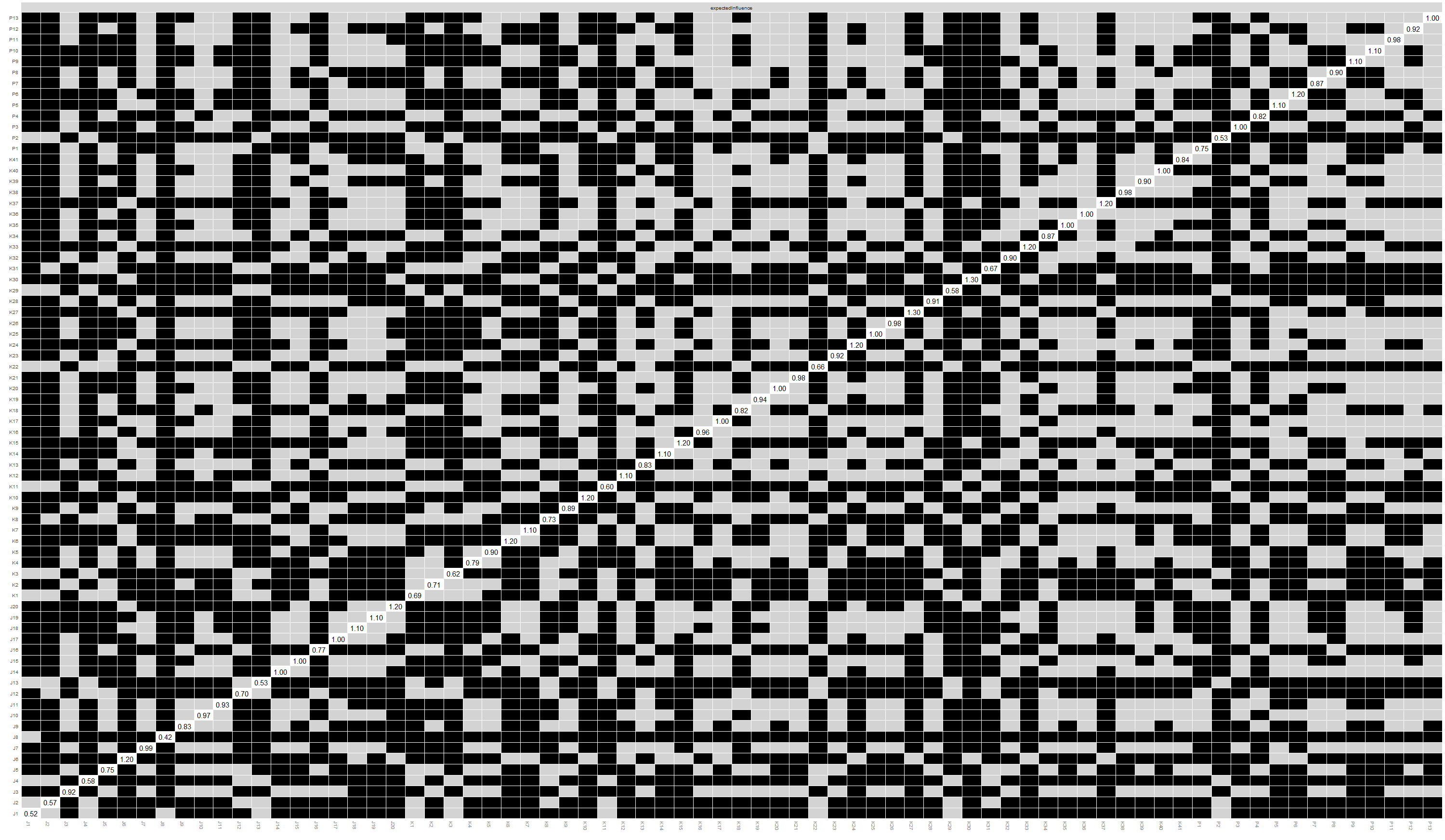


b.Wave 3

**Supplementary Figure 4.** The expectedInfluence difference tests for the depression-anxiety-PTSD network in children after COVID-19 of Wave 2 and Wave 3, respectively, based on 2500-bootstrapped bootnet results.

*Note*: Black box represents a node that differs significantly (α = 0.05) from another one in expectedInfluence and grey boxes signify no significant difference.


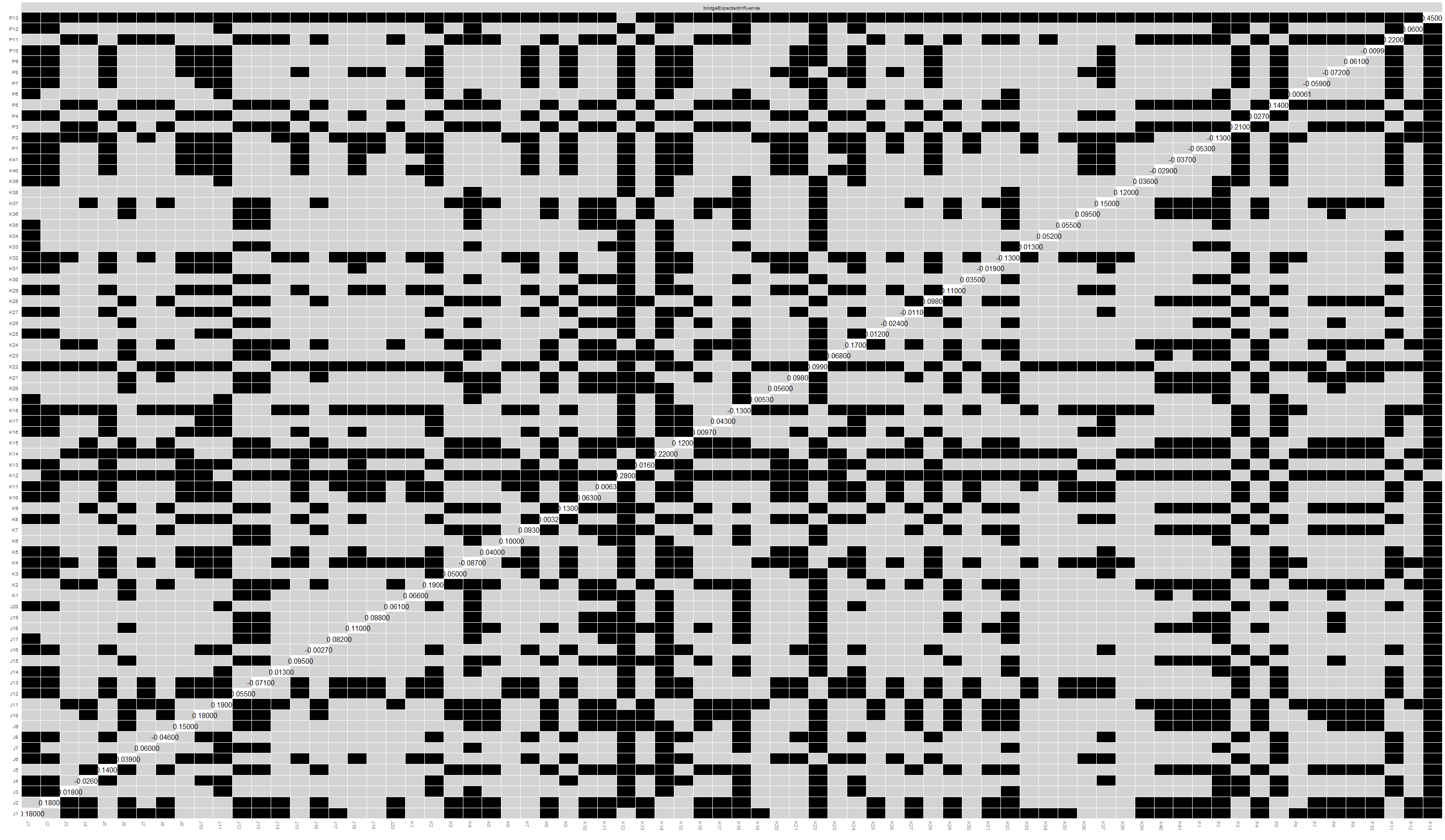


a.Wave 2


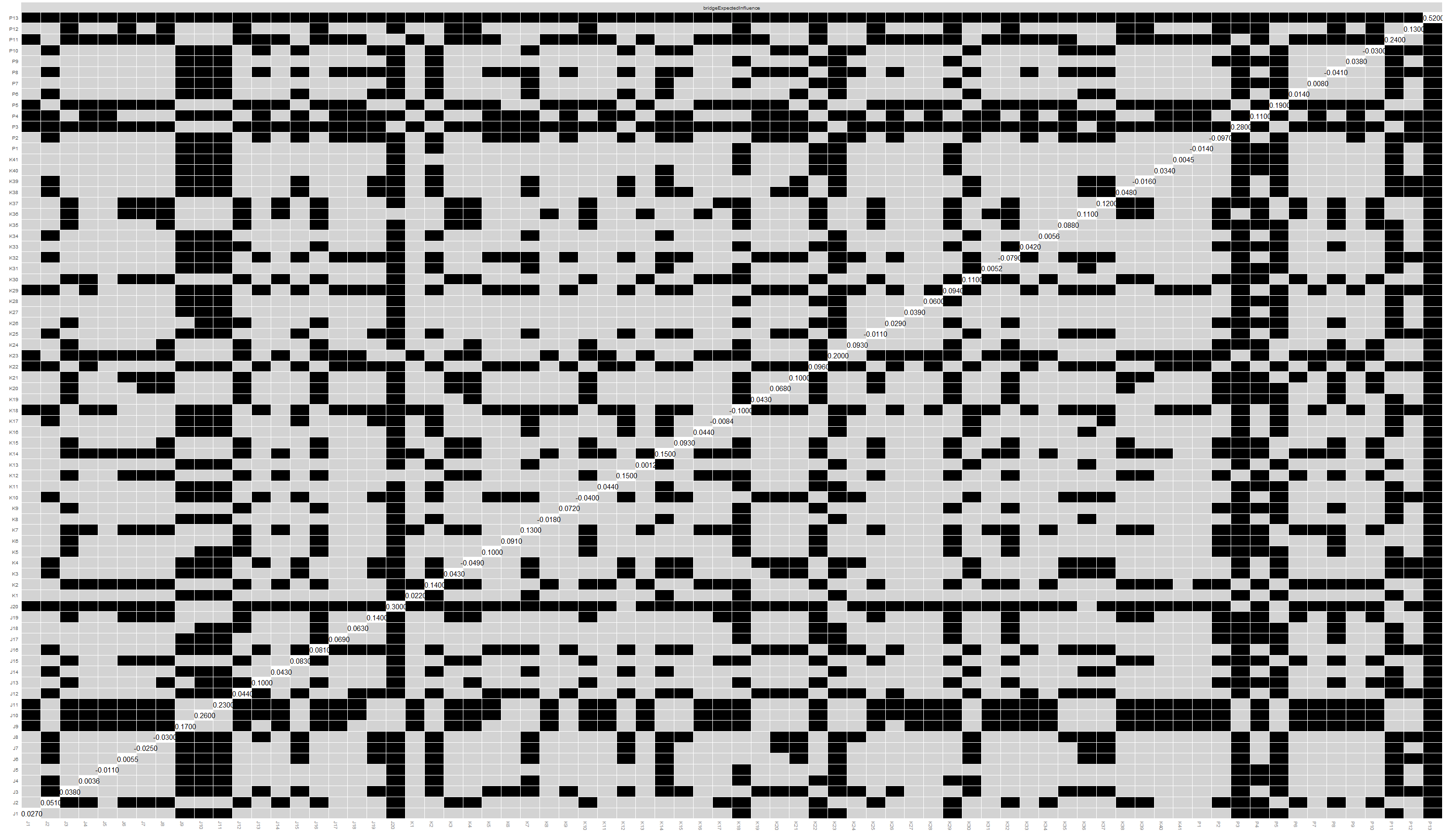


b.Wave 3

**Supplementary Figure 5.**  Bridge expectedInfluence difference tests for networks of the depression-anxiety-PTSD network in children after COVID-19 of Wave 2 and Wave 3, respectively, based on 2500-bootstrapped bootnet results.

*Note*: Black box represents a node that differs significantly (α = 0.05) from another one bridge expectedInfluence and grey boxes signify no significant difference.
